# The Composite Autonomic Symptom Score 31 Questionnaire: A Sensitive Test to Detect Risk for Autonomic Neuropathy

**DOI:** 10.1155/2023/4441115

**Published:** 2023-08-09

**Authors:** Sondre Meling, Erling Tjora, Heike Eichele, Niels Ejskjaer, Siri Carlsen, Pål Rasmus Njølstad, Christina Brock, Eirik Søfteland

**Affiliations:** ^1^Department of Medicine, Stavanger University Hospital, Stavanger, Norway; ^2^Department of Clinical Science, University of Bergen, Bergen, Norway; ^3^Children and Youth Clinic, Haukeland University Hospital, Bergen, Norway; ^4^Department of Biological and Medical Psychology, Faculty of Psychology, University of Bergen, Bergen, Norway; ^5^Regional Resource Centre for Autism, ADHD and Tourette Syndrome Western Norway, Division of Psychiatry, Haukeland University Hospital, Bergen, Norway; ^6^Department of Clinical Medicine, Faculty of Medicine, Aalborg University Hospital, Aalborg, Denmark; ^7^Steno Diabetes Center North Denmark, Aalborg University Hospital, Aalborg, Denmark; ^8^Department of Endocrinology, Aalborg University Hospital, Aalborg, Denmark; ^9^Mohn Center for Diabetes Precision Medicine, Department of Clinical Science, University of Bergen, Bergen, Norway; ^10^Mech-Sense, Department of Gastroenterology and Hepatology, Aalborg University Hospital, Aalborg, Denmark; ^11^Department of Medicine, Haukeland University Hospital, Bergen, Norway; ^12^Hormone Laboratory, Haukeland University Hospital, Bergen, Norway

## Abstract

**Background and Aims:**

Autonomic neuropathy is a common but often neglected complication of diabetes, prediabetes, and even in individuals with an elevated risk of diabetes. The Composite Autonomic Symptom Score (COMPASS) 31 is a validated and easy-to-use questionnaire regarding autonomic symptoms. We aimed to use a digitally, Norwegian version of the COMPASS 31 in people with different durations of diabetes and healthy controls to consider feasibility and to investigate if scores could discriminate between positive and negative outcomes for established tests for diabetic neuropathy, including cardiovascular autonomic neuropathy (CAN) and a novel method of examining the gastrointestinal visceral sensitivity.

**Method:**

We included 21 participants with longstanding type 2 diabetes, 15 with early type 2 diabetes, and 30 matched controls. The mean age for all groups was 69 years. Participants were phenotyped by cardiovascular autonomic reflex tests, electrical skin conductance, sural nerve electrophysiology, and the monofilament test. As a proxy for gastrointestinal visceral and autonomic nerve function, evoked potentials were measured following rapid rectal balloon distention.

**Results:**

Participants with longstanding diabetes scored a median (IQR) of 14.9 (10.8-28.7) points, early diabetes of 7.3 (1.6-15.2), and matched controls of 8.6 (4.1-21.6), *p* = 0.04. Women and men scored 14.4 (5.5-28.7) and 7.8 (3.6-14.6) points, respectively, *p* = 0.01. Participants with definite or borderline CAN scored 14.3 (10.4-31.9) points, compared to participants with no CAN, 8.3 (3.2-21.5), *p* = 0.04. Lowering the diagnostic cut-off from 16 to 10 points increased the sensitivity from 0.33 to 0.83, with a decreased specificity from 0.68 to 0.55.

**Conclusion:**

We successfully used COMPASS 31 in Norwegian. Thus, following the guidelines, we suggest clinical implementation for the assessment of autonomic neuropathy. Participants with longstanding diabetes had an increased likelihood of symptoms and signs of autonomic neuropathy. For screening purposes, the sensitivity was improved by lowering the cut-off to 10 points, with a lower score nearly excluding the diagnosis.

## 1. Introduction

Autonomic neuropathy is a common complication of diabetes mellitus and is associated with a wide range of symptoms, varying from mild to severe [1]. The condition is defined as a disorder of the autonomic nervous system in the setting of diabetes or metabolic derangements of prediabetes, after the exclusion of other causes, and may affect the cardiovascular, gastrointestinal, and genitourinary systems, as well as sudomotor function [2].

Guidelines from the American Diabetes Association recommend assessing symptoms and signs of autonomic neuropathy in type 2 diabetes, starting at the time of diagnosis [3]. Further, a 2010 expert consensus recommended screening for cardiovascular autonomic neuropathy (CAN) at the onset of type 2 diabetes, particularly if a history of poor glycaemic control or other known complications are present [2]. Assessment for CAN is also recommended before major surgery [4]. However, the lack of feasible tests and their demands in terms of time, resources, operator training, and patient preparations strongly limit the implementation of these guidelines. Hence, symptom-based questionnaires could represent a promising surrogate for the gold-standard CAN tests. Detecting early autonomic dysfunction would have implications for recommended treatment targets and interventions and aid symptom management.

The Composite Autonomic Symptom Score (COMPASS) 31 is a revised version of the 169-item Autonomic Symptom Profile assessing 11 domains of autonomic function, to the 31-item COMPASS, now assessing six domains: orthostatic hypotension, vasomotor, secretomotor, gastrointestinal, bladder, and pupillomotor functions. It is a validated, easy-to-use self-assessment instrument designed for clinical autonomic research and practice, which predicts CAN and diabetic polyneuropathy with fair diagnostic accuracy. It is also valid for evaluating treatment outcomes [5–7].

This study is aimed at exploring the use of a Norwegian, digitally distributed version of the COMPASS 31 in a present cohort with different diabetes durations and matched controls. We hypothesized that symptoms of autonomic neuropathy and objective findings of neuropathy were more prevalent in both longstanding diabetes and early diabetes compared to controls. We also hypothesized that the COMPASS 31 score correlated with established tests of diabetic neuropathy and plausibly with a novel method investigating rectal sensitivity and autonomic nerve conduction, assessed with rectally elicited evoked potentials.

## 2. Material and Methods

We conducted an observational case-control study, recruiting three groups: one group of people with type 2 diabetes for more than ten years (longstanding diabetes group), one with a newly diagnosed type 2 diabetes diagnosis within one year without using antidiabetic medication (early diabetes group), and controls matched for age, gender, and body mass index (BMI). The diagnosis was confirmed by performing an oral glucose tolerance test using criteria from the American Diabetes Association [8]. Exclusion criteria were major abdominal surgery, rectosigmoid disease interfering with sensitivity, chronic pancreatitis, uremic condition, atrial fibrillation or other major dysrhythmias, cardiac pacemaker, diabetic retinopathy, or present use of glucagon-like peptide 1 (GLP-1) receptor agonist or insulin.

The study was part of a larger project, the PanGut study, approved by the Regional Ethics Committee (REK Vest 2018#1790). Written consent in accordance with the Declaration of Helsinki was obtained from all participants before any study-related procedures. The relevant part of this study included three study days: one day of information, consent, and neuronal phenotyping; one day of oral glucose tolerance testing and basic blood samples; and one day of performing rapid rectal balloon distention. Lastly, COMPASS 31 was answered digitally at home. Recruitment and investigation were performed between September 2019 and December 2022, and all investigations were performed at a single centre (Bergen, Norway). Most of the participants were recruited through local newspaper advertising.

### 2.1. Examinations, Measures, and Variables

#### 2.1.1. Oral Glucose Tolerance Test

The test was performed after an overnight (10 h) fast with antidiabetic medications withdrawn for a total of three days, including the day of the examination. A cannula was placed in a cubital vein, with the forearm on the same side placed in a heating cuff to ensure arterialized blood. The participant ingested a 2–300 mL solution of 75 g anhydrate glucose. Blood glucose was measured before and 2 h after glucose ingestion using the HemoCue Glucose 201 DM RT (HemoCue, Angelholm, Sweden).

#### 2.1.2. COMPASS 31

The linguistically validated Danish version of COMPASS 31 was translated into Norwegian using a forward/backward translation method [9]. The questionnaire was distributed using the http://EasyTrial.net program (EasyTrial ApS, Aalborg, Denmark) and answered online at the participants' discretion. The maximum domain-specific weighted scores in the domains orthostatic, vasomotor, secretomotor, gastrointestinal, bladder, and pupillomotor were 40, 5, 15, 25, 10, and 5 points, respectively. The maximum total weighted score was 100 points. The recommended threshold supporting borderline CAN is 16 points [6].

#### 2.1.3. Neuronal Phenotyping

Alcohol consumption was not allowed for 24 hours before testing. Participants were instructed not to eat, use nicotine products, or drink caffeinated beverages within three hours before examinations. Medications were taken as normal, except for the use of stimulants or sedatives before the rapid rectal balloon distention test.

For cardiovascular reflex tests (CARTs) and short recordings of heart rate variability, we used the Vagus™ Device (Medicus Engineering, Aarhus, Denmark). CARTs were performed at rest, shortly after standing up, during deep breathing, and during the Valsalva manoeuvre. Blood pressure was measured following five minutes at rest, upon standing, and after one- and three-minute standing, using the WelchAllyn Connex ProBP 3400™ (EMEAI, Leiden, Netherlands). Orthostatic hypotension was defined as a decline in systolic blood pressure > 20 mmHg or in diastolic > 10 mmHg within three minutes of standing [10]. Stages of CAN were defined as borderline if one CART was abnormal, as definite if two or more CARTs were abnormal, and as severe if the latter was combined with orthostatic hypotension [2].

The Sudoscan™ Device (Impeto Medical, Paris, France) was used to test electrical skin conductance by measuring the chloride-ion flow produced by sweat glands in hands and feet following low-voltage electrical stimulation [11].

We used a 10 g monofilament to bilaterally pinprick the dorsum of each foot four times with the participant's eyes closed. Feeling 7-8 of 8 sensations was defined as no suspected diabetic peripheral neuropathy, 4-6 as possible, and ≤3 as likely [12].

Sural nerve conduction was tested using the point-of-care device NC-stat DPN Check™ (NEUROMetrix, Boston, USA). The device stimulates the sural nerve at the level of the ankle, recording the resulting responses on the calf [13].

#### 2.1.4. Visceral Sensitivity: Evoked Potentials following Rapid Balloon Distention in the Rectum

The equipment and protocol are described in detail elsewhere [14–17]. Electroencephalography (EEG) was recorded using a 64-channel extended 10-20 montage with reference electrode Fz. A rectal balloon was placed 15 cm above the anal verge. We recorded EEG continuously during two task conditions: elicitation of the earliest sensation and unpleasant threshold/feeling the urge to defecate, with 30 balloon pressure stimuli in each recording, respectively. A distinct and short stimulus was used with 150 ms of inflation, followed by instant deflation. A random interstimulus interval of 8 ± 2 seconds was enforced. EEG preprocessing and artefact reduction were done using independent component analysis in MATLAB (Mathworks, Natick, MA, USA). Data from the sensory-evoked potentials were pooled and analysed blindly.

#### 2.1.5. Data Analysis and Statistics

As the data represents secondary analyses, a formal power calculation was not feasible. Means ± standard deviations are used for data with a normal distribution, and medians with interquartile ranges for skewed data. Missing data were removed from the analysis. For parametric data, one-way ANOVA was used; for nonparametric data, we used the Kruskal-Wallis test for several independent samples and the Mann–Whitney *U* test for two samples. We used Spearman's rank-order test for correlation analysis. We calculated the area under the receiver operating characteristic curve (AUC), as well as sensitivity and specificity for diagnostic accuracy. Statistical significance was defined as a *p* value ≤ 0.05 for all analyses. Statistical analyses were performed using SPSS Version 28.0.1.0 (IBM, US).

## 3. Results

### 3.1. Subjects

We recruited a total of 66 participants (34 women), of whom 21 had longstanding type 2 diabetes, 15 had early type 2 diabetes (80% newly detected in the project), and 30 healthy controls. Baseline characteristics can be found in Table 1 and supplemental table 1.

### 3.2. COMPASS 31 Scores and Clinical Correlations

#### 3.2.1. Between-Group Differences

Participants with longstanding diabetes had a significantly higher COMPASS 31 score than both the group with early diabetes and the control group (*p* = 0.01, Table 2). The most contributing domains were the secretomotor and gastrointestinal.

#### 3.2.2. Scores Influenced by Medications

There was an association between those with longstanding diabetes using dipeptidyl peptidase 4 (DPP-4) inhibitors and a higher total score (rho -0.319, *p* = <0.01), score in the secretomotor domain (rho -0.248, *p* = 0.05), and score in the gastrointestinal domain (rho -0.333, *p* = <0.01), with a significantly different score in the gastrointestinal domain (Table 3). Other medications with known gastrointestinal side effects or known to affect the autonomic nervous system had no significant influence on COMPASS 31 scores.

#### 3.2.3. Sex Differences

Women scored higher than men on the total COMPASS 31 score and in all domains except for bladder function. The domains contributing the most were secretomotor and gastrointestinal (Table 4). Women with longstanding diabetes had the highest median score of 24.3 points.

### 3.3. Neuronal Phenotyping and COMPASS 31 Score

Two participants had definite CAN based on CARTs: one in the early diabetes group and one in the control group. Definite or borderline CAN was detected in 31% of participants with longstanding diabetes, 23% with early diabetes, and 17% of controls, *p* = 0.54. Based on the monofilament test, the prevalence of possible or likely peripheral neuropathy was 29% (longstanding diabetes), 13% (early diabetes), and 6.7% (controls), *p* = 0.04 for longstanding diabetes compared to controls. A total of 14 participants (five in the longstanding group, two in the early group, and seven in the control group) did not complete the CARTs, most of them (12) not being able to perform an adequate Valsalva manoeuvre, the last two probably due to poor skin contact.

No associations were found within the respective groups when comparing CARTs and heart rate variability with COMPASS 31 scores, but independently of groups, scores correlated with definite or borderline CAN, rho = 0.283, *p* 0.04. The score difference was also significant (Table 5). No significant associations were detected between the total COMPASS 31 score or different domain scores and the sudomotor function test score, monofilament test, or sural nerve function test. Further, no significant associations could be detected between COMPASS 31 scores and rectal sensitivity or evoked brain potentials.

All results from CARTs, heart rate variability, sudomotor function, sural nerve check, monofilament test, and rapid rectal balloon distention tests can be found in supplemental files (S2-4) or in a previous publication from the present study [17].

**Figure 1:**
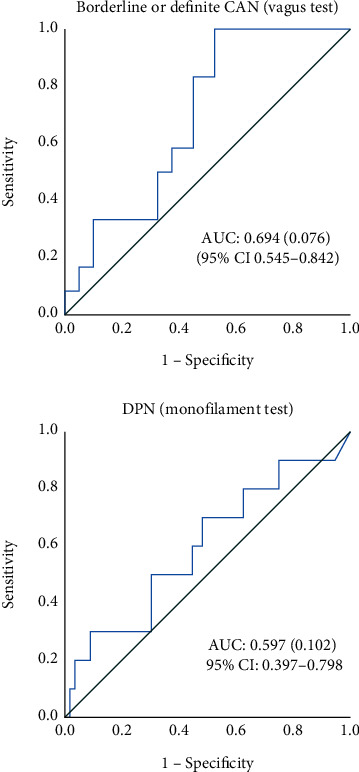
ROC curves for CAN (Vagus test) and DPN (monofilament test).

Using a cut-off for a total COMPASS 31 score of 16 points for CAN in our population, the sensitivity was 0.33 and the specificity was 0.68. Changing the cut-off to 10 points increased the sensitivity to 0.83 with a specificity of 0.55 (Table 5 and Figure 1).

## 4. Discussion

We successfully used the Norwegian version of COMPASS 31. More symptoms and signs associated with autonomic neuropathy were reported in those with longstanding diabetes than in people with early diabetes and controls. The results were partly influenced by DPP-4 inhibitors, mostly in the gastrointestinal domain. Women reported more symptoms than men. Independently of diabetes status, there were clinical correlations between increased symptom burden and borderline or definite CAN. No significant correlations were detected with other established neuropathy tests or with the novel test of evoked potential following rapid rectal balloon distention or rectal sensitivity.

### 4.1. COMPASS 31, Ease of Use

Symptoms and signs of autonomic neuropathy should lead to further testing, but until recently, questionnaires regarding symptoms and signs have not been validated [2, 4]. Since being revised to COMPASS 31 in 2012, the questionnaire has been validated in different languages and used in several research trials, including diabetic neuropathies [5–7, 9, 18–21]. In our experience, the questionnaire was easy to use, and digital distribution and answering were feasible, despite our participants' rather high average age. We, therefore, find the practical aspects of digital questionnaire handling suitable for clinical studies, making large-scale real-life studies feasible. In our opinion, symptoms of autonomic neuropathy are a neglected area in terms of care for people with diabetes, and implementation studies using this questionnaire on a larger scale should be performed. We did not experience issues that would limit the application of the questionnaire in an individual clinical setting.

### 4.2. Symptoms of Autonomic Neuropathy, a Higher Burden in Longstanding Diabetes and Women

People with longstanding diabetes had a higher COMPASS 31 score than the groups with early diabetes and controls. This is in line with other studies on autonomic symptoms in diabetes, indicating that the duration of diabetes is a risk factor [2, 22]. Thus, contrary to previous studies showing that autonomic dysfunction could be present already at prediabetic stages, and also negating our hypothesis, we did not detect this either by CARTs or by COMPASS 31 [4]. However, a challenge when screening for small fibre neuropathy at the early stages of diabetes is that it can often be asymptomatic [2, 22]. To our knowledge, no other study on COMPASS 31 included a group of early diabetes. A different questionnaire, the Survey of Autonomic Symptoms, has been validated for detecting autonomic symptoms in early diabetic neuropathy, but with inclusion criteria that make the study incomparable to ours [23].

The gastrointestinal domain contributes the most to the higher total score in longstanding diabetes. This supports the former knowledge that patients with diabetes experience more gastrointestinal symptoms than people without diabetes. Symptoms are often diverse, ranging from mild to life-threatening, and despite a high prevalence in outpatient clinics (≥70%), they are often unrecognized by clinicians [24]. The underlying mechanisms for diabetic gastroenteropathy may include structural remodelling of the gut wall, dysfunctional autonomic regulation, biochemical dysfunction, immune-mediated alterations, and inflammatory properties within the enteric nervous system [25–27]. We did not uncover any impact on COMPASS 31 scores by medications to have known gastrointestinal side effects, but surprisingly, there was a slightly higher total score in people using DPP-4 inhibitors, driven by the gastrointestinal domain. The DPP-4 inhibitor mostly used was sitagliptin (80%). Earlier studies in people with diabetes using sitagliptin have reported a marginally elevated risk of gastrointestinal adverse events vs. placebo (5.0% vs. 4.6 and 1.8% vs. 1.4%) [28, 29]. Our study was not powered to detect such differences, and the results may be due to other causes, such as confounding by indication (i.e., more people with diabetes who had gastrointestinal symptoms due to other causes may fail on metformin and/or GLP-1 receptor agonists and hence receive DPP-4 inhibitors).

Women scored higher in total, and in all domains, except for bladder symptoms. The most significant sex differences were found in the secretomotor and gastrointestinal domains. The secretomotor domain contains a question regarding the degree of sweating, which could be explained by the remaining symptoms of menopause. Women did not use more medications that are related to an increase in any of the symptoms reported. Regarding the higher score for women in the gastrointestinal domain, this has also been previously reported in a population-based study, with one of the reasons proposed for this related to a higher prevalence of gastrointestinal functional disorders in women [30]. The same study also suggested that the negative effect diabetes exerts on daily life is more pronounced in women, as a possible explanation. Epidemiologic studies have reported a higher prevalence of gastrointestinal symptoms in women, regardless of having diabetes or not [30, 31]. The results could also reflect that men may generally underreport symptoms, which have been proposed for other conditions, such as self-reporting in depression [32].

### 4.3. Correlation between COMPASS 31 Score and Other Tests

We did not uncover any correlations between continuous scores in COMPASS 31 and CARTs, sudomotor function testing, sural nerve function, or the monofilament test. Other studies have reported correlations between COMPASS 31 and CARTs, especially for deep breathing, lying to standing, and for some parameters of heart rate variability [18, 20]. One possible explanation could be the limited cases of definite CAN in the present population. Despite an average of 17 years since diagnosis, our longstanding diabetes population had few microvascular complications, acceptable values for HbA1c, and a near-normal average BMI. The reasons for this may partly be explained by excluding people using GLP-1 analogue and/or insulin and people with retinopathy, the first mentioned because of other aspects of the PanGut study investigating the incretin effect. Both obesity in type 2 diabetes and retinopathy have been correlated with CAN [2, 33]. The specific characteristics of our study population are of importance concerning external validity.

We could also not detect any correlation between COMPASS 31 scores, including the gastrointestinal domain, and rectal sensitivity or evoked potentials following rapid balloon distention in the rectum. This might support other studies reporting a lack of correlation between symptoms, especially regarding diabetic gastroenteropathies, and objective findings such as motility disturbances in the gastrointestinal tract [34]. Other aspects of the results from the rapid balloon distention test are published elsewhere [17].

### 4.4. Comparison to Studies Validating COMPASS 31

Our reported prevalence of 31% borderline CAN in the group with longstanding diabetes is comparable to other studies validating the questionnaire reporting a prevalence of 29-36% (although these included 13-14% with definite CAN as well) [6, 18]. The mentioned studies display higher scores than our study; the differences probably reflect the different populations, with the referenced studies all recruiting from diabetes clinics or other tertiary centres, some reporting higher HbA1c, and some reporting a higher prevalence of known microvascular disease. For comparison, our group of longstanding diabetes was small, included people with a near normal HbA1c and BMI, and had few microvascular complications.

### 4.5. COMPASS 31 as a Screening Tool

COMPASS 31 is considered a well-validated screening tool for autonomic dysfunction and small nerve fibre neuropathy, both independently and in combination with other tests [2, 18, 19, 21]. As there were no associations between COMPASS scores within each group for borderline/definite CAN but a correlation for the score with the groups combined, we merged the groups for further diagnostic considerations. This seems highly relevant as our groups had a substantial overlap in glycaemia from the OGTT, with our overall population ranging from a mild phenotype of longstanding diabetes to a control group that also included people with prediabetes. Using the recommended threshold score of 16 points, we found a particularly poor sensitivity (0.33) for borderline/definite CAN. However, by reducing the cut-off to 10 points, sensitivity increased markedly (0.83) with only a slight decrease in specificity (0.68 to 0.55) and a high negative predictive value (0.92). Of interest in this regard, Treister et al. also reported a cut-off of 10 being optimal for screening purposes, with a sensitivity of 93% and specificity of 38% for small fibre polyneuropathy, confirmed by epidermal nerve fibre density [19]. In contrast to this, another study, which reported a prevalence of 17% for confirmed CAN, recommended a cut-off score of 28.7 points for definite CAN. Though this study had a population of 89% with borderline or definite CAN, compared to ours, it had higher values for HbA1c and BMI and a higher total score, mainly because of a higher score in the orthostatic domain [35].

### 4.6. Methodological Considerations

COMPASS 31 is yet to be formally validated in the Norwegian language. Still, with the high similarity between written Danish and Norwegian languages, as well as similar cultures and demographics, we argue that the risk of biases in the Norwegian version is low [9]. A forward-backward translation was performed, and no discrepancies were detected. Internal validity for subjects with diabetes and CAN or peripheral neuropathy is reported as acceptable in a comparable country (Italy) [6, 18]. We consider it a strength that the questionnaires were provided digitally, as it could increase the probability of providing a more unbiased answer.

When performing the tests for neuronal phenotyping, we did not enforce the discontinuation of any medications. Several drugs could impact autonomic function tests; however, short-term discontinuation could also influence results, e.g., rebound tachycardia discontinuing betablockers, so we opted to leave them unchanged [36].

We acknowledge a limitation regarding our selected nature of participants, with a mean age of 69 years and near normal weight. Still, especially regarding age, we find the present cohort less investigated, but with an increasing prevalence of type 2 diabetes, probably due to a generally increased life expectancy.

Finally, we acknowledge the few participants in every group, thus the lack of statistical power. Comparable studies validating COMPASS 31 have found a sample size between 60 and 90 participants adequate [6, 18].

## 5. Conclusion

We found a Norwegian, digitally distributed version of COMPASS 31 easy to use and evaluate, and believe it is feasible for both research in larger groups and clinical practice. In the present cohort, higher COMPASS 31 scores were associated with definite or borderline CAN, with longstanding diabetes, and with female sex, but not with results from other tests for diabetic neuropathy or the novel test investigating evoked potential after rectal balloon distention or with rectal sensitivity. In screening for people with early autonomic dysfunction, we propose a cut-off of 10 points, considering further CAN diagnostics if the patient scores above this level.

## Figures and Tables

**Table 1:** Clinical characteristics at baseline.

Clinical characteristics	Longstanding diabetes *n* = 21	Early diabetes *n* = 15	Controls *n* = 30	*p* value
Age (years at recruitment)	68.9 ± 7.8	69.3 ± 5.5	69.5 ± 6.2	0.950
Gender (women/men)	10/11	8/7	16/14	0.911
BMI (kg/m^2^)	26.5 ± 4.4	25.7 ± 4.1	25.5 ± 3.8	0.680
Diabetes duration (years)	16.8 ± 4.9	0	0	n/a
Fasting glucose (OGTT), mmol/L	9.4 ± 2.1	7.2 ± 1.0	6.0 ± 0.6	<0.001
2-hour glucose (OGTT), mmol/L	18.7 ± 3.9	13.1 ± 4.2	7.9 ± 1.5	<0.001
HbA1c (mmol/Mol) (%)	54 ± 11.2 (7.1)	43 ± 4.9 (6.1)	37 ± 3.0 (5.5)	<0.001
Total cholesterol (mmol/L)	4.2 ± 0.8†	4.5 ± 1.2†	5.5 ± 1.0	<0.001
HDL (mmol/L)	1.3 ± 0.3†	1.4 ± 0.4†	1.9 ± 0.5	<0.001
LDL (mmol/L)	2.4 ± 0.6†	2.8 ± 1.1	3.3 ± 0.8	0.001
Triglycerides (mmol/L)	1.7 ± 1.3†	1.3 ± 0.5	1.0 ± 0.4	0.009
eGFR (ml/min/1.73 m^2^)	84.9 ± 13.5	82.3 ± 11.7	80.3 ± 12.3	0.458
Systolic blood pressure rest (mmHg)	135 ± 15††	152 ± 14	139 ± 20††	0.015
Diastolic blood pressure, rest (mmHg)	80 ± 6††	86 ± 7	81 ± 7	0.023
Comorbidity (*N*)				
Nephropathy	0	0	0	n/a
Distal neuropathy, %	4.8	6.7	0	0.400
Hypertension, %	52	47	17	0.017
Cardiovascular disease, %	4.8	13	3.3	0.401
Drugs (*N*)				
Metformin, %	81	0	0	n/a
Sulphonylurea, %	19	0	0	n/a
DPP-4 inhibitor, %	48	0	0	n/a
SGLT2 inhibitor, %	38	0	0	n/a
Another antidiabetic medication, %	9.5	0	0	n/a
Diet-treated diabetes, %	9.5	0	0	n/a
Betablocker, %	4.8	20	13	0.370
ACE-I/ARB, %	48	40	10	<0.001
Other antihypertensive medication, %	19	13	7	0.410
Lipid modifying treatment, %	67	47	13	<0.001
Smoking status,% (present/past/never)	10/38/52	7/13/80	3/43/54	0.300

Data are means ± SD unless otherwise indicated. *p* values using one-way ANOVA or Pearson's chi square test. Post hoc test for continuous data between groups using Bonferroni: †significant compared to controls, ††significant compared to early diabetes, all other groups were <0.001 with significant difference to each other. Diabetes duration, comorbidity, smoking status, and drugs are self-reported. Abbreviations: BMI: body mass index; OGTT: oral glucose tolerance test; HDL: high-density lipoprotein; LDL: low-density lipoprotein; eGFR: estimated glomerular filtration rate; DPP-4: dipeptidyl peptidase-4; SGLT-2: sodium-glucose cotransporter 2; ACE-I: angiotensin converting enzyme inhibitor; ARB: angiotensin receptor blocker.

**Table 2:** COMPASS 31 score for groups.

	Group score, points				
	Longstanding diabetes	Early diabetes	Control	*p* value	All groups
Orthostatic	0.0 (0.0-14.0)	0.0 (0.0-0.0)	0.0 (0.0-0.0)	0.23	0.0 (0.0-12.0)
Vasomotor	0.0 (0.0-0.0)	0.0 (0.0-0.0)	0.0 (0.0-0.0)	0.76	0.0 (0.0-0.0)
Secretomotor	4.3 (0.0-6.4)†	0.0 (0.0-0.0)	2.1 (0.0-4.3)	0.03	1.1 (0.0-4.8)
Gastrointestinal	5.4 (2.7-8.9)	1.8 (0.0-6.3)	2.7 (0.0-7.1)	0.06	3.6 (0.9-7.1)
Bladder	1.1 (0.0-2.8)	1.1 (0.0-2.2)	1.1 (0.0-2.2)	0.81	1.1 (0.0-2.2)
Pupillomotor	1.0 (0.3-2.0)	1.0 (0.0-2.0)	1.0 (0.0-1.7)	0.49	1.0 (0.0-1.7)
Total weighted	14.9 (10.8-28.7)††	7.3 (1.6-15.2)	8.6 (4.1-21.6)	0.04	11.9 (4.5-21.6)

Data are medians with interquartile range, *p* values comparing all three groups using the Kruskal-Wallis test. †Significant in pairwise comparison to early diabetes. ††Significant in pairwise comparison to both other groups. For pairwise comparison, the Mann–Whitney test was used.

**Table 3:** COMPASS 31 score for the group with longstanding diabetes, with and without DPP-4 inhibitors.

	With DPP-4 inhib. *n* = 10	Without DPP-4 inhib. *n* = 11	*p* value
Secretomotor	4.3 (0.0-7.0)	2.1 (0.0-6.4)	0.65
Gastrointestinal	6.7 (5.8-10.5)	3.6 (1.8-5.4)	0.02
Total weighted	17.7 (11.4-35.8)	14.9 (7.3-20.8)	0.43

Data are medians with interquartile range, *p* values using the Mann–Whitney test.

**Table 4:** COMPASS 31 score for different sex.

	Sex		*p* value
	Women	Men	
Orthostatic	0.0 (0.0-13.0)	0.0 (0.0-3.0)	0.40
Vasomotor	0.0 (0.0-0.0)	0.0 (0.0-0.0)	0.14
Secretomotor	4.3 (0.0-6.4)	0.0 (0.0-2.1)	<0.01
Gastrointestinal	5.8 (1.5-9.8)	1.8 (0.0-4.5)	<0.01
Bladder	1.1 (0.0-2.2)	1.1 (0.0-2.2)	0.87
Pupillomotor	1.0 (0.0-2.1)	1.0 (0.0-1.7)	0.86
Total weighted	14.4 (5.5-28.7)	7.8 (3.6-14.6)	0.01

Data are medians with interquartile range, *p* values using the Mann–Whitney test.

**Table 5:** COMPASS 31 score for definite/borderline CAN, no CAN, and results from monofilament test, for the entire population.

	COMPASS 31 score	AUC (95% CI)	Cut off: 16 p. Sensitivity PPV	Specificity NPV	Cut off: 10 p. Sensitivity PPV	Specificity NPV
CARTs						
Definite/borderline CAN (*n* = 12)	14.3 (10.4-31.9)	0.69 (0.55-0.84)	0.33 0.24	0.68 0.78	0.83 0.36	0.55 0.92
No CAN (*n* = 40)	8.3 (3.2-21.5)					
*p* value	0.04					
Monofilament test						
Possible/likely DPN (*n* = 10)	15.5 (4.4-32.3)	0.60 (0.40-0.80)	0.40 0.23	0.70 0.87	0.70 0.29	0.50 0.83
No DPN (*n* = 56)	11.0 (4.3-21.8)					
*p* value	0.33					

Data are medians with interquartile range. *p* values using the Mann–Whitney test. The area under the curve (AUC) is estimated for predicting diagnostic accuracy. PPV = positive predictive value; NPV = negative predictive value; CARTs = cardiovascular reflex tests; CAN = cardiovascular autonomic neuropathy; DPN = diabetic peripheral neuropathy. Definite/borderline CAN = one or more pathological CARTs. Possible/probably DPN with <six sensations on monofilament test. A total of 14 participants did not complete the CARTs, five in the longstanding group, two in the early group, and seven in the control group.

## Data Availability

The participants of this study did not give written consent for their data to be shared publicly, so due to the sensitive nature of the research, supporting data is not available.

## Supplementary Materials

Supplementary Materials S1 Additional comorbidity, S2: neuronal phenotyping, S3: amplitude and latency (mean average) from evoked potentials following rapid balloon distention in the rectum, S4: values for latencies and amplitudes for evoked potentials
