# Supplementary material for: The Composite Autonomic Symptom Score 31 Questionnaire: A Sensitive Test to Detect Risk for Autonomic Neuropathy
Source: J Diabetes Res. 2023 Aug 9;2023:4441115. doi: 10.1155/2023/4441115 (PMC10432092; doi:10.1155/2023/4441115)
Supplement: Supplementary Materials — S1 Additional comorbidity, S2: neuronal phenotyping, S3: amplitude and latency (mean average) from evoked potentials following rapid balloon distention in the rectum, S4: values for latencies and amplitudes for evoked potentials [file 4441115.f1.docx]

**Supplemental files/tables – Changes highlighted in blue**

**NEW: S1: Other comorbidity**

|  | Longstanding diabetes n=21 | Early diabetes  n=15 | Controls  n=30 |
| --- | --- | --- | --- |
| Attention deficit and hyperactivity disorder | 1 |  |  |
| Chronic diarrhoea | 1 |  |  |
| Paroxysmal atrial fibrillation, or former ablation |  | 2 |  |
| Arthritis |  | 1 | 2 |
| Deep venous thrombosis | 1 |  |  |
| Obstructive sleep apnoea | 2 | 1 | 1 |
| Essential tremor |  |  | 1 |
| Hiatus hernia |  | 1 | 1 |
| Gout | 1 |  |  |
| Heart valve surgery | 1 |  |  |
| Appendectomy | 1 |  |  |
| Hip prothesis | 1 |  | 1 |
| Asthma | 2 |  | 1 |
| Removed malignant melanoma | 2 |  | 2 |
| Post viral fatigue | 1 |  | 1 |
| Gastritis/Oesophagitis |  | 2 | 2 |
| Treated testicular cancer | 1 | 1 | 1 |
| Mild anal incontinence |  |  | 1 |
| Treated ovary/uteri cancer | 2 |  | 1 |
| Hypothyroidism | 3 | 3 | 4 |
| Osteoporosis/osteopenia | 1 | 2 | 5 |
| Peripheral neuropathy | 1 |  |  |
| Obstipation |  |  | 1 |
| Neuroborreliosis |  |  | 1 |
| Irritable bowel syndrome |  |  | 3 |
| Ischialgia |  |  | 1 |
| Performed mastectomy |  |  | 1 |
| Migraine | 1 |  | 1 |
| Inflammatory bowel disease | 1 |  |  |
| Nephrolithiasis | 1 |  |  |
| Benign prostate hyperplasia | 1 |  | 1 |
| Surgery on frontoethmoidal mucocele. |  | 1 |  |
| Surgery shoulder | 1 |  |  |
| Treated small intestinal cancer |  | 1 |  |
| Treated prostate cancer |  | 2 |  |
| African sleeping sickness (Trypanosomiasis) |  | 1 |  |
| Sjögrens syndrome | 1 |  | 2 |
| Obesity |  |  | 2 |
| Performed parathyroidectomy |  |  | 1 |
| Knee prothesis |  |  | 1 |
| Pericarditis |  |  | 1 |
| Cholecystectomy |  |  | 1 |
| Rheumatoid arthritis |  |  | 1 |
| Inguinal hernia |  |  | 1 |
| Former spinal stenosis | 1 |  |  |
| Former acromegaly |  |  | 1 |
| Urinary incontinence |  |  | 2 |
| Allergy |  |  | 1 |
| Ulcerous colitis |  | 1 |  |
| Tinnitus |  |  | 1 |
| Postmenopausal syndrome |  |  | 1 |
| Urinary retention |  |  | 1 |
| Fatigue |  |  | 1 |

Legend: Reported as numbers (n). Data are self-reported by the participants.

**S2: Neuronal phenotyping**

| Parameters | Longstanding diabetes  n=21 | Early diabetes  n=15 | Controls  n=30 | P-values |
| --- | --- | --- | --- | --- |
| **Rectal sensation**  Pressure VAS 1, kPa  Pressure VAS 5, kPa  **CARTs and HRV**  HR, bpm  SDNN, ms  RMSSD, ms  LF, ms^2^  HF, ms^2^  Total, ms^2^  R/S ratio (n=61)  Abnormal R/S ratio, %  E/I ratio (n=59)  Abnormal E/I ratio, %  VM ratio (n=52)  Abnormal VM ratio, %  *CAN*  *No/borderline/definite*, %  Orthostatic hypotension, %  Severe/advanced CAN, %  **Sural nerve check**  DPN, Velocity, m/s (n=64)  DPN, Amplitude, µV  *Peripheral neuropathy: No/mild/mod./serious, %*  **Monofilament test**  Felt pinpricks (n=66)  *Peripheral neuropathy: Unlikely/possibly/likely, %*  **Sudomotor function**  Hands, µSiemens (n=65)  *Normal/mod. reduced /severely reduced, %*  Feet, µSiemens (n=66)  *Normal/mod. reduced /severely reduced, %* | 3.7+1.1*  145+50  68.5+8.3  29.5 (19.6-46.7)  18.1 (10.8-45.6)  58.5 (33.7-145.4)  69.7 (13.6-152.0)  247.2 (121.9-600.6)  1.08 (1.03-1.12)  6.3  1.18 (1.06-1.31)  25  1.41 (1.33-1.65)  0  69/31/0  14  0  46.5 (43-50)  6.5 (5-9)  76/10/10/0  8 (6-8)*  71/24/5  65.8+14.4  70/25/5  73.6+12.4  71/19/10 | 4.0+1.3*  147+58  64.0+8.0  32.7 (17.0-38.3)  19.5 (11.0-26.2)  93.9 (25.7-126.8)  63.4 (15.5-104.1)  306.3 (134.9-469.5)  1.09 (1.04-1.15)  6.7  1.14 (1.07-1.23)  20  1.41 (1.32-1.48)  6.7  77/15/8  20  6.7  47 (40.2-51)  5 (4-9)  80/13/7/0  8 (8-8)  87/13/0  67.2+14.1  67/33/0  77.7+6.7  80/20/0 | 3.0+0.9  150+50  63.1+11.6  29.1 (21.5-37.5)  21.2 (9.6-31.8)  77.9 (34.5-155.5)  44.1 (13.0-99.4)  324.2 (161.9-586.8)  1.08 (1.06-1.15)  6.7  1.13 (1.11-1.23)  6.7  1.47 (1.33-1.62)  3.3  83/13/4  17  0  48 (43.5-52)  7 (5-9)  87/0/7/3  8 (8-8)  93/0/7  71.3+15.2  77/20/3  75.3+13.5  87/7/7 | <0.01  0.94  0.15  0.94  0.96  0.97  0.99  0.72  0.38  0.96  0.73  0.34  0.33  0.55  0.54  0.90  0.325  0.56  0.54  0.68  0.08  0.06  0.39  0.81  0.60  0.45 |

Legend: Results of neuronal phenotyping given as mean +SD or median (IQR). Asterisk (*) indicates statistically significant differences compared to controls. HR = mean heart rate, bpm = beats per minute, during 5 min. rest, SDNN = standard deviation from the mean heartbeat interval value (net effect of the autonomic regulation), RMSSD = root mean square of the standard deviation (activity level of the parasympathetic regulation). LF = low frequency activity (represents sympathetic tone), HF = high frequency activity (represents parasympathetic tone), TP = total power (power spectrum of RR intervals throughout the frequency ranges − net autonomic function). R/S ratio: 30:15 Ratio = ratio between maximum HR within the first 15 s after standing up and minimum HR within the first 30 s after standing up (predominantly parasympathetic test). E/I-ratio = mean ratio between the longest and shortest RR-interval during deep respiration (measures of baroreflex sensitivity and capacity — predominantly parasympathetic tests). VM ratio: ratio between maximum heart rate at the end of forced expiration and minimal heart rate during inspiration-expiration in rest after appr. 30 seconds after releasing pressure (predominantly sympathetic and baroreflex mediated test). Reference cardiovascular reflex tests (CARTs) ratios are age adjusted. CAN =cardiovascular autonomic neuropathy. CAN was predicted as borderline if one abnormal CART ratio, and definite if two or more abnormal ratios. Orthostatic hypotension is defined as a decline in systolic blood pressure of > 20 mmHg or diastolic > 10 mmHg within three minutes of standing. Severe/advanced CAN is defined as the combination of definite CAN and orthostatic hypotension. Risk of diabetic peripheral neuropathy (DPN) performing the sural nerve check is defined by the software. For the monofilament test feeling 7-8 of 8 sensations is defined as unlikely DPN, feeling 4-6 as possible DPN, and feeling 3 or less, as likely DPN. Stages of sudomotor function are also defined by the software. P-values from categorical outcomes from Chi Square test.

**S3: Amplitude and latency (mean average) from evoked potentials following rapid balloon distention in the rectum**

a)


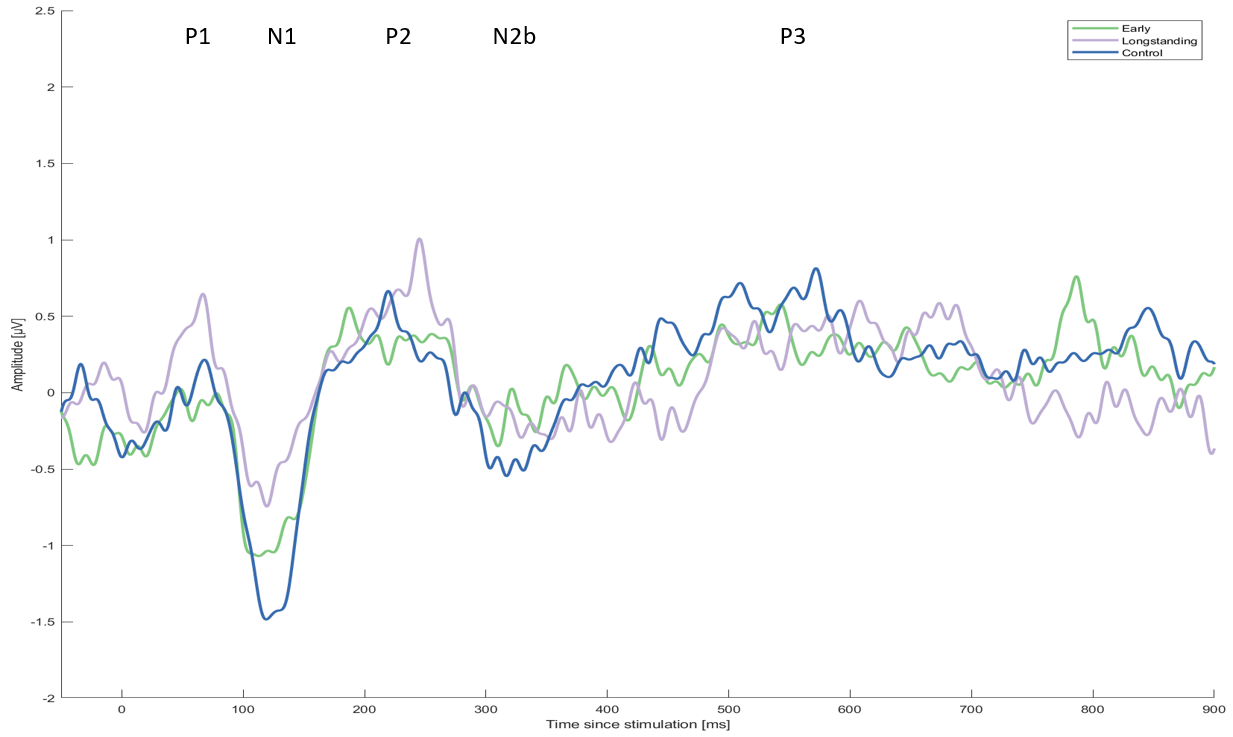


b)


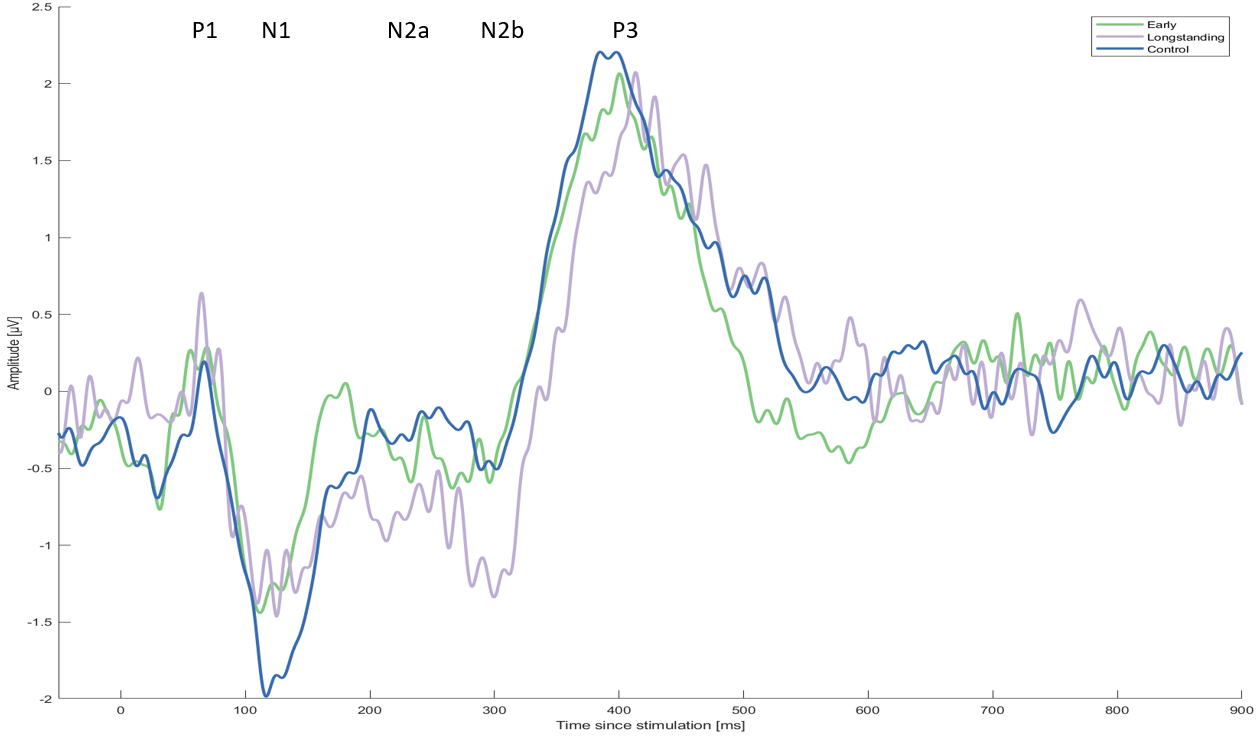


c)


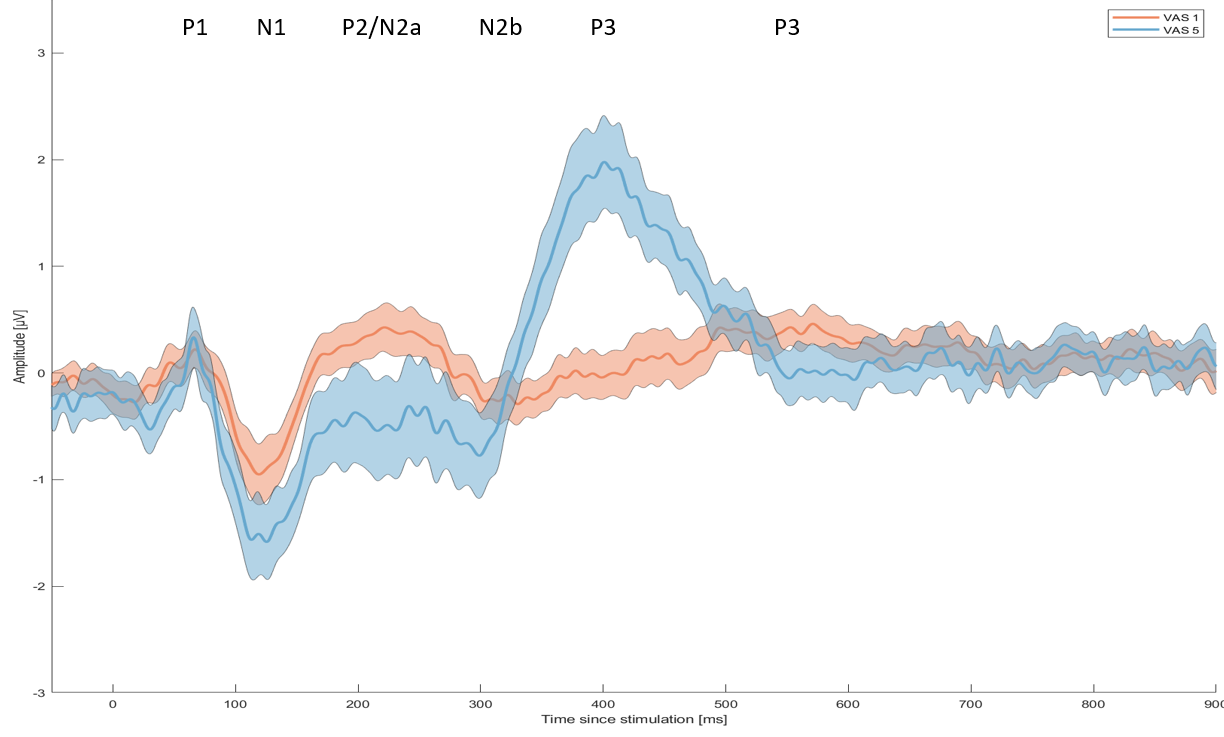


Amplitude and latency for VAS 1 (a) and VAS 5 (b) respectively, for the three groups and total VAS 1 and VAS 5 means for all observations (c). P1 indicates the first positive peak, N1 the first negative peak, etc elicited by rapid balloon distention. Signals occurring <250 ms (P1-2, N1-2a) represent stimulus-specific exogenous processing, providing information on the sensitivity of the visceral afferent pathways, while >250 ms (N2b and P3) typically involve later cognitive processes. As expected, N2b and P3 in VAS 5 have shorter latency and larger amplitude due to the increased salience/stimulus intensity compared to VAS 1.

**S4**: **Latencies and amplitudes for evoked potentials**

| VAS 1-amplitude  P1  N1  P2  N2b  P3  VAS 1- latency  P1  N1  P2  N2b  P3  VAS 5 -amplitude  P1  N1  N2b  N2a  P3  VAS 5-latency  P1  N1  N2a  N2b  P3 | Longstanding diabetes  1.6+1.4  -2.0+1.2  1.8+1.1  -1.8+0.8  1.6+1.0  64.1+22.0  125.2+31.9  221.5+58.6  353.8+87.3  518.2+55.6  1.4+1.4  -2.9+2.1  -2.6+2.2  0.25+2.6  3.1+1.7  64.1+29.7  131.8+36.6  230.9+50.6  297.3+42.7  417.7+43.1 | Early  diabetes  0.7+0.6  -1.9+1.0  1.3+1.5  -1.3+0.8  1.5+1.5  56.7+25.3  115.3+26.7  210.4+54.2  308.3+61.6  487.9+85.9  0.8+0.8  -2.4+1.4  -1.8+1.6  1.2+1.6  2.7+1.9  59.6+14.1  113.5+21.4  193.4+54.9  285.3+50.5  405.8+22.3 | Controls  1.1+1.0  -2.3+1.0  1.6+1.1  -1.5+1.1  1.7+0.9  52.4+24.4  114.8+17.2  220.0+61.4  327.0+56.8  513.8+62.5  0.7+0.9  -2.8+1.3  -1.4+1.6  1.0+1.4  3.0+1.5  54,7+14.4  122.5+24.9  217.1+46.6  283.5+36.3  398.5+29.8 | p-value  0.082  0.422  0.403  0.387  0.758  0.317  0.361  0.844  0.170  0.363  0.084  0.567  0.074  0.297  0.733  0.760  0.204  0.116  0.520  0.155 |  |
| --- | --- | --- | --- | --- | --- |

Data are in means +SD, p-values from one-way ANOVA, between all groups. Latencies are in milliseconds, amplitude in microvolt. Abbreviations: VAS = visual analogue scale. VAS 1 = earliest sensation. VAS 5 = unpleasant threshold. P1 indicates the first positive peak, N1 the first negative peak, etc.
